# Effect of Preoperative N-Acetylcysteine on Postoperative Blood Loss Parameters in Cardiac Surgery Patients

**DOI:** 10.1155/2011/859020

**Published:** 2011-06-21

**Authors:** Amber R. Wesner, Marcia L. Brackbill, Christine S. Sytsma

**Affiliations:** Pharmacy Department, Winchester Medical Center, 1840 Amherst Street, Winchester, VA 22601, USA

## Abstract

*Purpose*. To determine if recent preoperative exposure to n-acetylcysteine (NAC), Mucomyst, increases postoperative blood loss in cardiac surgery patients. *Methods*. Retrospective review of cardiac surgery patients who underwent a cardiac catheterization within four days of surgery and whose serum creatinine was ≥1.0 mg/dL. The study groups were those who received NAC in the pericatheterization period versus those who did not. The primary endpoint was postoperative chest tube output at 24, 48, and 72 hours. Secondary endpoints included number of transfusions and other bleeding parameters. *Results*. Mean blood loss in the first 24 hours was 962 ± 595 mL in the treatment group (*n* = 79) and 1,178 ± 788 mL in the control group (*n* = 106), *P* = .040. Blood loss between groups at 48 (366 ± 318 mL versus 412 ± 363 mL, *P* = .382) and 72 (194 ± 300 mL versus 176 ± 224 mL, *P* = .643) hours was not significantly different. There were no significant differences in postoperative transfusions or other bleeding parameters. *Conclusions*. Preoperative exposure to NAC did not increase postoperative blood loss or negatively affect other bleeding parameters.

## 1. Introduction

Contrast agents used for diagnostic procedures such as cardiac angiography can lead to contrast-induced nephropathy [1]. N-acetylcysteine (NAC), Mucomyst, an amino acid derived from cysteine, causes vasodilation, increases glutathione production, and has antioxidant properties all of which may reduce or prevent this kidney damage [1–3]. Currently, evidence is mixed as to the effectiveness of NAC at preventing contrast-induced nephropathy. Several studies, including Tepel et al. and the APART trial, found NAC to be an effective treatment to prevent renal damage from contrast media [1–5]. However, results showing NAC to be ineffective at preventing contrast-induced nephropathy have also been found [6–8]. 

Despite the conflicting evidence concerning NAC's effectiveness, it is often used prior to contrast agents due to its low cost and relatively benign side effect profile. NAC administration has also been studied intraoperatively due to its potential to help reduce inflammation and ischemia-reperfusion organ damage associated with cardiac surgery [9]. However, recently NAC's effect on blood loss and coagulation has been questioned [9, 10]. Studies have found that intraoperative use of NAC impairs postoperative hemodynamics resulting in: increased blood loss, increased transfusions, and increased risk of reoperation [9, 10]. Whether NAC use in the perioperative setting may also contribute to potential increases in blood loss is not known. However, it is necessary to evaluate NAC in this population, as many patients undergo coronary angiography immediately prior to cardiac surgery.

The primary objective of this study was to determine the effects of preoperative NAC exposure on postoperative chest tube blood loss at 24, 48, and 72 hours after cardiac surgery. Secondary objectives included the effect of recent NAC exposure on first postoperative PT, hemoglobin (Hgb), hematocrit (Hct), platelets, and number of postoperative blood transfusions in the first 72 postoperative hours. 

## 2. Materials and Methods

This retrospective chart review was conducted at a regional 411-bed not for profit community hospital. Data were collected from patients' medical records accessed through the medical records department of the hospital. Patients were included for chart review if they presented for cardiac surgery between June 1, 2006 and June 1, 2009, were >18 years old, had a precardiac catheterization serum creatinine (Scr) measurement of ≥1.0 mg/dL, and underwent cardiac catheterization within four days of cardiac surgery. Patients undergoing emergent cardiac surgery were excluded.

Patients in the NAC treatment arm received oral NAC 600 mg twice daily for two days, beginning the day before cardiac catheterization, for kidney protection associated with contrast agents. NAC had to have been administered within four days of cardiac surgery to be included in this study. Patients in the NAC group also received saline hydration and may have received concomitant bicarbonate therapy. Patients in the control group received saline hydration and may have received bicarbonate therapy prior to cardiac catheterization.

The primary outcome was blood loss between groups measured as chest tube output at 24, 48, and 72 hours after surgery in patients who received NAC within 4 days of cardiac surgery compared to those who did not. Secondary outcomes measured were first postop PT time, lowest Hgb, lowest Hct, and platelet count at 24, 48, and 72 hours after surgery, number of postop blood transfusions, and length of hospital stay.

The time period utilized for data collection was specifically chosen as this was a period in time where cardiac catheterization patients were all receiving the same peri-procedural protocol with regards to medications. Thus, all patients who met inclusion criteria during the designated study period were screened for potential study inclusion. Statistical analysis was completed using SPSS (SPSS statistics, version 17). Demographic data were analyzed using the *t*-test and Chi Square where appropriate. Preoperative characteristics and primary and secondary objectives were analyzed using the *t*-test. A *P* value <.05 was considered significant. 

This study was approved by the hospital's Institutional Review Board. 

## 3. Results and Discussion

### 3.1. Results

**Figure 1:**
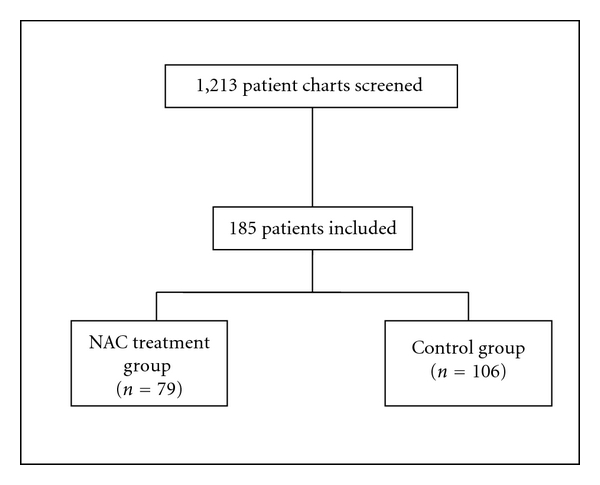
Study arms.

One thousand two hundred thirteen charts were screened for potential study inclusion, and one hundred eighty-five met inclusion criteria. Seventy-nine patients comprised the NAC treatment group, and one hundred six patients comprised the control group. See Figure 1.

 Demographic data are presented in Table 1. Groups were similar at baseline with the exception of history of diabetes, history of hypertension, and type of cardiac surgery. Patients in the NAC group were more likely to have a history of diabetes and hypertension while patients in the control group were more often CABG only procedures.

Preoperative profiles are presented in Table 2. Patients treated with NAC had higher serum creatinine measurements prior to cardiac catheterization and before surgery. NAC-treated patients also had significantly lower hemoglobin and hematocrit levels prior to surgery.

Primary endpoint data are presented in Table 3. Patients treated with NAC lost less blood in the first 24 hour postoperative period. Blood loss in the first 24 hours was 962 ± 577 mL in the NAC treatment group and 1,178 ± 788 mL in the control group which was statistically significant (*P*-value =.040). Blood loss between the treatment and control groups was not significantly different during the second and third 24 hour periods.

Secondary endpoints are presented in Table 4. No significant differences were observed between groups. 

### 3.2. Discussion

Because of NAC's antioxidant properties, it has been evaluated for its potential to decrease inflammation during surgery [10]. However, several studies have found the use of intraoperative NAC to be associated with increased blood loss and need for transfusions [9, 10]. Little is known about the mechanism of this potential impairment in hemostasis, and it is also unknown whether preoperative exposure to NAC will also put patients at risk for increased blood loss. The present study found that NAC, within 4 days of cardiac surgery, was not associated with increased blood loss measured as chest tube output. Patients treated with NAC lost less blood in the first 24 hours after surgery than patients who did not receive preoperative NAC. This study also showed similar blood loss between groups at 48 and 72 hours after surgery, similar PT results, hemoglobin, hematocrit, platelet counts, number of transfusions, and length of hospital stay suggesting that NAC did not negatively affect hemostasis.

Study patients were similar at baseline. However, more patients in the NAC group had a history of diabetes and hypertension. These conditions may have worsened patient's kidney function thus indicating the need for prophylactic use of NAC. Also, patients treated with NAC had higher serum creatinine levels prior to cardiac catheterization and cardiac surgery. This difference between groups is understandable given those patients with elevated serum creatinine are those in need of protection from further nephropathy and therefore receive NAC. The mean change in serum creatinine from baseline to time of discharge was not significantly different between groups *P* = .769. There was a 0.011 ± 0.211 mg/dL mean change in serum creatinine for the NAC group and an 0.004 ± 0.175 mg/dL mean change in the control group. Thus, indicating NAC was effective in preventing contrast-induced nephropathy in these patients. In addition, patients treated with NAC had significantly lower hemoglobin and hematocrit levels before surgery, but this did not translate into lower hemoglobin and hematocrit levels postoperatively or an increased number of transfusions.

These results differ from previous studies, and several factors may have contributed to these findings. In studies that have found an association between NAC and increased blood loss, NAC was given intravenously during surgery compared to the population in this study who received oral NAC prior to cardiac surgery. Both Wijeysundera et al. and Niemi et al. utilized IV bolus and IV infusions for NAC administration whereas patients in our study received NAC orally. Also, in the two studies by Wijeysundera et al. and Niemi et al., NAC was administered in the immediate preoperative period [9, 10]. The study by Wijeysundera et al. administered an NAC bolus immediately following induction of anesthesia and then began a continuous NAC infusion which ran during cardiac surgery and for 4 hours following the end of cardiopulmonary bypass [9]. Niemi et al. administered NAC also as a bolus immediately following anesthesia induction followed by an NAC continuous infusion for 24 hours [10]. This is in contrast to our study where patients could have received NAC anytime within 4 days of cardiac surgery. Whether this difference in route and timing of NAC administration accounts for the difference in bleeding outcomes is unknown. The dosage of NAC also varied between studies. Larger doses were used in the studies which found NAC to be associated with increased bleeding loss. Wijeysundera et al. studied a dose of 100 mg/kg given IV over 30 minutes followed by an infusion of 20 mg/kg/hr which was continued until 4 hours after cardiopulmonary bypass, and Niemi et al. gave a 150 mg/kg bolus over 20 minutes followed by 150 mg/kg infusion which was continued for 24 hours [9, 10]. Both of the previous studies found an association between NAC use and increased blood loss. These doses are in contrast to this study where patients received 600 mg twice daily the day before and the day of cardiac catheterization, and this study was not associated with increased blood loss. However, whether NAC's effect on hemostasis is dose related is not known and could present an area for further study.

This study has several limitations. The study was a retrospective chart review and thus is limited to previously recorded data. This may have introduced bias due to incomplete documentation. Also, our sample size was small, and the time frame for use of NAC in the preoperative setting is not well established in the current literature. It is possible that our results may have changed if we had limited the preoperative NAC dosage window to less than 4 days. The difference in route of administration and dose of NAC administered between this study and previous studies also must be considered when interpreting these results. This study investigated the oral administration of NAC, at 600 mg twice daily, as is the protocol at our institution which did not match the intravenous weight-based dosing used in the studies which found an association between NAC and increased bleeding. Future studies should prospectively investigate further whether larger doses of NAC, intravenous administration, or length of time from NAC administration till surgery affects blood loss. In addition, there were some differences in baseline characteristics between our groups. Patients treated with NAC were more likely to have a history of diabetes, history of hypertension, and elevated serum creatinine. Although it is to be expected that patients treated with NAC have baseline renal insufficiency compared to patients not selected to receive this therapy, these were still statistically significant differences between groups. 

Due to these limitations and based on having several unanswered questions regarding NAC and blood loss, such as differences between routes, doses, and time period till cardiac surgery, NAC should be studied further to assess its affects on blood loss and the safety of its use in the peri- and intraoperative period. 

## 4. Conclusions

Preoperative exposure to NAC did not increase postoperative blood loss in the first 72 postoperative hours in the cardiac surgery patient. Furthermore, NAC did not negatively affect PT time, hemoglobin, hematocrit, platelets, or number of transfusion requirements. 

## Figures and Tables

**Table 1:** Patient demographics.

Variable	N-acetylcysteine group (*n* = 79)	Control group (*n* = 106)	*P *Value
Age in years^a^	67.3 ± 9.1	65.5 ± 10.8	.233
Gender			.456
Males	72.2%	79.2%	
Females	27.8%	20.8%	
Baseline ADP inhibitor use	5.6%	4.7%	.451
Baseline aspirin use	92.4%	96.2%	.508
History of diabetes	58.2%	35.8%	.007
History of dyslipidemia	83.3%	82.1%	.451
History of hypertension	86.1%	75.5%	.029
Type of surgery			.001
CABG only	70.9%	85.8%	
Valve only	6.3%	5.7%	
CABG + Valve	7.6%	4.7%	
Other^b^	15.2%	3.8%	
Height (cm)^a^	172 ± 9.9	172 ± 9.0	.952
Weight (kg)^a^	88.9 ± 22.9	88.5 ± 19.1	.908

^a^Mean ± standard deviation.

^b^Transmyocardial revascularization (TMR) or MAZE procedure.

CABG: coronary artery bypass graft.

ADP: adenosine diphosphate.

**Table 2:** Preoperative (Pre-op) characteristics.

Endpoint	N-acetylcysteine group (*n* = 79)	Control group (*n* = 106)	*P* Value
Days between cath and surgery	2.05 ± 1.2	1.91 ± 1.1	.398
Precath Scr (mg/dL)	1.7 ± 1.5	1.2 ± 0.3	<.005
Preop Scr (mg/dL)	1.6 ± 1.4	1.2 ± 0.4	.002
Preop PT time (secs)	10.7 ± 1.1	10.5 ± 0.9	.284
Preop Hgb (g/dL)	12.3 ± 1.6	13.2 ± 2.0	.001
Preop Hct (%)	36.6 ± 4.3	39.2 ± 5.2	<.005
Preop platelets (×10^3^ mm^3^)	201 ± 54	209 ± 66	.372
Received precath hydration ≥ 75 mL/hr	22	25	
Received precath bicarb	5	2	
Received precath hydration and bicarb	1	0	

Cath: catheterization.

Scr: serum creatinine.

PT: prothrombin time.

Hgb: hemoglobin.

Hct: hematocrit.

**Table 3:** Primary Endpoints.

Endpoint	N-acetylcysteine group (*n* = 79 )	Control group (*n* = 106)	*P* Value
Chest tube output in first 24 hours (mL)	962 ± 577	1178 ± 788	.040
Chest tube output in second 24 hours (mL)	366 ± 318	412 ± 363	.382
Chest tube output in third 24 hours (mL)	194 ± 300	176 ± 224	.643

**Table 4:** Secondary Endpoints.

Endpoint	N-acetylcysteine group (*n* = 79)	Control group (*n* = 106)	*P* Value
First postop PT time (secs)	15.9 ± 3.2	16.6 ± 3.6	.196
Lowest Hgb in first 24 hrs (g/dL)	8.4 ± 1.3	8.8 ± 1.6	.088
Lowest Hgb in second 24 hrs (g/dL)	8.7 ± 1.1	8.9 ± 1.1	.227
Lowest Hgb in third 24 hrs (g/dL)	9.0 ± 1.0	9.2 ± 1.0	.132
Lowest Hct in first 24 hrs (%)	25.0 ± 3.7	26.0 ± 4.7	.075
Lowest Hct in second 24 hrs (%)	25.9 ± 3.3	26.6 ± 3.3	.161
Lowest Hct in third 24 hrs (%)	26.8 ± 3.2	27.6 ± 3.2	.100
Lowest platelet count in first 24 hrs (×10^3^/mm^3^)	116 ± 43	114 ± 35	.735
Lowest platelet count in second 24 hrs (×10^3^/mm^3^)	122 ± 44	116 ± 39	.283
Lowest platelet count in third 24 hrs (×10^3^/mm^3^)	139 ± 50	127 ± 49	.121
Number of postop transfusions	2.2 ± 2.5	2.3 ± 2.4	.653
Postop hospital stay (days)	8.8 ± 8.8	7.6 ± 5.1	.252

PT: prothrombin time.

Hgb: hemoglobin.

Hct: hematocrit.
